# Combining CNN Features with Voting Classifiers for Optimizing Performance of Brain Tumor Classification

**DOI:** 10.3390/cancers15061767

**Published:** 2023-03-14

**Authors:** Nazik Alturki, Muhammad Umer, Abid Ishaq, Nihal Abuzinadah, Khaled Alnowaiser, Abdullah Mohamed, Oumaima Saidani, Imran Ashraf

**Affiliations:** 1 Department of Information Systems, College of Computer and Information Sciences, Princess Nourah bint Abdulrahman University, P.O. Box 84428, Riyadh 11671, Saudi Arabia; 2 Department of Computer Science & Information Technology, The Islamia University of Bahawalpur, Bahawalpur 63100, Pakistan; 3 Faculty of Computer Science and Information Technology, King Abdulaziz University, P.O. Box. 80200, Jeddah 21589, Saudi Arabia; 4 Department of Computer Engineering, College of Computer Engineering and Sciences, Prince Sattam Bin Abdulaziz University, Al-Kharj 11942, Saudi Arabia; 5 Research Centre, Future University in Egypt, New Cairo 11745, Egypt; 6 Department of Information and Communication Engineering, Yeungnam University, Gyeongsan 38541, Republic of Korea

**Keywords:** brain tumor prediction, healthcare, deep convolutional features, ensemble learning

## Abstract

**Simple Summary:**

This study presents a hybrid model for brain tumor detection. Contrary to manual featur extraction, features extracted from a convolutional neural network are used to train the model. Experimental results show the efficacy of CNN features over manually extracted features and model can detect brain tumor with a 99.9% accuracy.

**Abstract:**

Brain tumors and other nervous system cancers are among the top ten leading fatal diseases. The effective treatment of brain tumors depends on their early detection. This research work makes use of 13 features with a voting classifier that combines logistic regression with stochastic gradient descent using features extracted by deep convolutional layers for the efficient classification of tumorous victims from the normal. From the first and second-order brain tumor features, deep convolutional features are extracted for model training. Using deep convolutional features helps to increase the precision of tumor and non-tumor patient classification. The proposed voting classifier along with convoluted features produces results that show the highest accuracy of 99.9%. Compared to cutting-edge methods, the proposed approach has demonstrated improved accuracy.

## 1. Introduction

Medical image analysis is a growing field that uses a variety of modern image processing techniques. As a result, a variety of diseases can now be detected in a timely manner. Early detection can help in the treatment of most life-threatening diseases such as tumors, eye disease, Alzheimer’s, blood clots, and cancer [1]. Biopsies and images of the infected areas are used inr the diagnosis of these life-threatening diseases. Images of the affected areas are typically used to diagnosing diseases in the early stages. Biopsies on the other hand are used to confirm the presence of certain diseases [2]. In such cases, it is crucial that the modeling of infected areas is highly accurate and easily visualized.

The brain is a critical organ in the human body and plays a vital role in controlling the body and decision-making. Therefore, brain tumors are life-threatening conditions. Most malignancies involve the nervous system and thus it has important implications regarding diagnosis. The brain parenchyma, also referred to as metastases, is commonly involved [3]. The majority of brain tumors are brain metastases, which are estimated to have an incidence rate 10 times higher than primary brain tumors [4]. There are different types of gliomas and their malignancies also vary. In addition, they are different from most common primary brain tumors such as meningiomas and pituitary adenomas. The timely diagnosis and detection of primary brain tumors are essential as they are cancerous and life-threatening. The proper treatment of these cancerous tumors is critical, and different techniques are available to treat them. Treatment plans for brain tumors depend on how early the diagnosis is made and on the tumor type. Different diagnostic techniques are available tor efficiently diagnose brain tumors, such as magnetic resonance imaging (MRI) [5]. MRI provides vital information to classify the brain tumor and helps in treatment decisions [6].

The early detection of a brain tumors increases survival chances. Manual diagnosis and detection are laborious, time-consuming, and faulty. Expert radiologists are frequently required to gain a better understanding, identify the tumor, and compare tumor tissues to those in neighboring locations. For medical image analysis computer-aided imaging technology helps in the brain tumor early detection and categorization. The use of the latest technologies for the identification of brain tumors saves time and manpower as well. MRI is currently the most often used non-invasive technique for detecting brain tumors [7]. MRI scanning is the most commonly used technique for brain analysis. MRI can observe the difference in soft tissues which makes MRI advantageous over other techniques for brain tumor diagnosis. It has no side effects because it does not involve the application of ionizing radiation to brain areas [8]. MRI technique is extensively used by radiologists because it has the ability to diagnose the abnormal growth of cells. For brain tumor detection, a dual-channel DC-BTD system was proposed by Zahoor et al. [9]. The authors used MRI images that show how minimal false negatives are. They used the static S-shaped features and for the discriminant dynamic features they used the D-channel. The study also included the use of techniques such as data normalization, augmentation, and four distinct machine learning classifiers. The findings of the study showed better results with 98.70% accuracy than existing studies. Similarly, ref. [10] used ensemble models to classify and diagnose brain tumors by enhancing MRI images with an average filter. Deep learning models are used for feature extraction such as ResNet-18 and AlexNet. SoftMax and SVM were used to classify these features. The proposed hybrid approach AlexNet+SVM achieved an accuracy score of 95.10%. Daz-Pernas et al. [11] used MRI images for the classification of brain tumors. They did not perform the pre-processing in their study. Their proposed approach achieved an accuracy of tumor classification of 97.3%. In addition to the MRI scanning, all the imaging techniques produce images in greyscale, except for the color Doppler technique, which produces color images. However, other techniques for tissue segmentation regions such as post-processing do not produce the desired results [12,13].

Many studies focus on the use of deep learning models for brain tumor detection. For example, an intelligent deep learning-based system for brain tumor detection was designed by Khan et al. [7]. They classified brain tumors into three classes: Pituitary, Meningioma, and Glioma. The proposed system is HDL2BT (Hierarchical Deep Learning Based Brain tumor) which utilises CNN to classify brain tumours in an exact and precise manner. The proposed model shows a precision of 92.13%. A deep learning-based system DeepTumorNet was designed by Raza et al. [14] for the categorization of the three different kinds of brain tumors, the same used by the [7]. CNN GoogLeNet architecture was utilized as the base of the system. The authors tested the system on the publicly available dataset and achieved good results. They acquired an accuracy score of 98.67%. Ahmad et al. [15] used a number of classical classifiers together with different transfer learning-based deep learning approaches to detect brain tumors. The authors used seven approaches for transfer learning including Xception, ResNet50, InceptionResNetV2, VGG-16 and VGG-19, DenseNet201, and InceptionV3. These transfer learning models were followed by machine learning models. The findings of the study showed an accuracy of 98.39%.

Various researchers have used transfer learning models and achieved robust results for the identification of brain tumors [16,17]. Amran et al. [16] designed a hybrid deep tumor network for brain tumor detection by combining a CNN with GoogleNet. The author achieved 98.91% accuracy using Inceptionresnetv2 in [17]. The study [18] suggested a conditional segmentation strategy based on a residual network, as well as an attention approach based on an extreme gradient boost. The results showed that the CNN-CRF-Resnet system achieved an accuracy of 99.56% across all three classes. Samee et al. designed a hybrid transfer learning system GN-AlexNet for the classification of brain tumors and achieved an accuracy of 99.51% [19].

An ensemble deep learning-based system was designed by Rasool et al. [20] for the categorization of three different kinds of brain tumors. The authors used the ensemble deep learning model with fine-tuned GoogleNet and achieved an accuracy of 93.1%. As opposed to that, when the authors used GoogleNet as a feature extractor they obtained an accuracy of 98.1%. As genetic mutation is the primary reason for brain cancer, classifying and segmenting brain tumors using genomic information can help in diagnosis [21]. Using AI approaches, it is possible to identify disease-related molecular features from radiological medical images by assessing the genomic state of genetic mutations on numerous genes and cell proteins [22,23]. Authors combined AI with radio genomics for brain tumor detection in [24].

Some studies utilized the same dataset used in this study and have shown promising results. The study [25] employs an ensemble learning approach based on machine learning to detect brain cancers. NGBoost classifier was used alongside ETC, RF, GBC, and ADA for comparison. The findings revealed that the use of NGBoost produced a significantly higher accuracy of 98.54%. Aryan Sagar Methil [26] presented a deep learning approach for detecting brain tumors. Several image processing techniques were applied for obtaining better results. The employed CNN model achieves an accuracy of 95%. Shah et al. [27] utilized MR scans to determine the prognosis of brain malignancies. They proposed a refined EfficientNet-B0 for brain tumor prediction and also employed data augmentation techniques to obtain higher-quality photos. The proposed Efficient-B0 system acheived an accuracy of 98.87%. However, the proposed transfer learning model was a complex neural network model that required millions of parameters to train, which was the key drawback of the study.

This paper aims to develop a simple machine learning-based system that uses CNN as the feature engineering technique to classify patients with brain tumors and normal patients using MRI scan data. In summary, the proposed system offers the following advantages

- This study proposes an ensemble model that utilizes convolutional features from a customized CNN model for predicting brain tumors. The proposed ensemble model is based on logistic regression and a stochastic gradient descent classifier with a voting mechanism for making the final output.
- The impact of the original features is analyzed against the performance of models using convolutional features.
- The performance comparison is performed using various machine learning models including random forest (RF), K-nearest neighbor (k-NN), logistic regression (LR), gradient boosting machine (GBM), decision tree (DT), Gaussian Naive Bayes (GNB), extra tree classifier (ETC), support vector machine (SVM), and stochastic gradient descent (SGD). Moreover, the performance of the proposed model is compared with leading-edge methodologies in terms of accuracy, precision, recall, and F1 score.

The remaining sections are arranged as follows. Section 2 discusses the proposed system’s components and functions. Section 3 provides the results, whereas Section 4 contains the discussions and conclusion.

## 2. Materials and Methods

The ’brain tumor’ dataset used for the detection of the disease, the proposed approach, and the steps taken for the proposed framework is discussed in this section. The machine learning classifiers utilized in this work are also briefly described in this section.

### 2.1. Dataset

For the performance comparison, various machine learning models were utilized in this study. The selection of the right dataset is a vital step, this study makes use of the “Brain tumor” dataset which is publicly available on Kaggle [28]. The dataset contained 3762 instances, 13 features, and a target class. Of these 13 features, 5 were first-order features and 8 were texture features. The first-order features were the standard deviation, mean, kurtosis, variance, skewness, and texture features are entropy, contrast, homogeneity, energy, dissimilarity, correlation, coarseness, and ASM (Angular second moment). The target features contained two classes: tumors and non-tumor. Of 3762 instances, 2079 belonged to the non-tumor class and 1683 belonged to the tumor class.

### 2.2. Machine Learning Models

In this work, nine machine learning algorithms were utilized to identify brain tumors including RF, SVM, k-NN, LR, GBM, DT, GNB, ETC, and SGD. A brief explanation of these machine-learning models is given here.

#### 2.2.1. Random Forest

RF [29,30] is a well-known and widely used tree-based machine learning algorithm. From the previous random vector, RF generates the independent random vector and distributes them among all the trees. It is a step-by-step process in which the root node divides the data into its child nodes, and so on until the leaf nodes are reached. In RF, each node of the tree independently classifies the feature’s objective variables, and after that class votes. The classification results from the decision trees depend on the majority voting. Error in RF is calculated using the following formula

(1) $$\begin{array}{c} PE^{*}=P_{\left(i,j\right)}\left(f\left(i,j\right)<0\right) \end{array}$$

where random vectors are represented by the *i* and *j* and these random vectors represent the probability. And *f* computes the average number of votes across all random vectors for the desired outcome [31], it is calculated as

(2) $$\begin{array}{c} f\left(i,j\right)=av_{K}I\left(H\left(i\right)=j\right)-max_{y\neq j}av_{K}I\left(h_{k}\left(i\right)=y\right) \end{array}$$

#### 2.2.2. Decision Tree

A DT is one of the tree-based methods used for the classification of brain tumors. It handles classification and regression problems efficiently [32,33]. The major issue in DT is the finding of the root node at each level. Attribute selection is the method used to identify the root node. “Gini Index” and “information gain” are the attribute selection techniques. The following formula may be used to compute the Gini value.

(3) $$\begin{array}{c} \mathrm{Gini}=1-\sum_{i=1}^{classes}p{\left(i|t\right)}^{2} \end{array}$$

Impurity in the dataset is calculated using Gini. The other method used for attribute selection is information gain. It calculates the purity of the dataset. Information Gain for each attribute can be calculated using the following steps

**Step 1:** determine the target’s entropy.
**Step 2:** compute each attribute’s entropy.

The following formula may be used to get the entropy for a collection of instances *D*.

(4) $$\begin{array}{c} \mathrm{entropy}\left(D\right)=\sum_{i=1}^{\left|c\right|}P_{r}\left(C_{i}\right)log_{2}P_{r}\left(C_{i}\right),\mathrm{where}\sum_{i=1}^{\left|c\right|}P_{r}\left(C_{i}\right)=1 \end{array}$$

For the construction of the trees in all the tree-based classifiers in this work information gain and the Gini index value are used.

#### 2.2.3. K-Nearest Neighbour

k-NN is the first choice for medical data mining. k-NN is a straightforward instance-based classifier [34,35]. A supervised learning model called k-NN compares new data to existing cases to determine how similar they are, then groups the new data with those cases that have the highest similarity. Finding the similarity of the data involves measuring the distance between the new and existing data points. For distance calculation, various methods are available such as Manhattan, Euclidean, Murkowski, etc. Although k-NN is utilized for regression problems it is widely used to solve classification problems. There are multiple parameters in k-NN and these need to be correctly refined for good results.

#### 2.2.4. Logistic Regression

LR is a supervised learning-based machine learning classifier that is statistics-based [36,37,38]. The input characteristics (X: input) can be categorized by LR into a discrete set of target values (Y: output). A logistic function is employed in LR to determine the likelihood of either class 0 or class 1. A logistic function typically has the shape of an “S” as in the equation below.

(5) $$f\left(x\right)=\frac{L}{1+e^{-m(v-v_{o})}}$$

LR uses the sigmoid function for probability prediction. The following formula can be used to determine the sigmoid function.

(6) $$\sigma \left(x\right)=e^{x}\left(e^{x}+1\right),\sigma \left(x\right)=1\left(1+e^{-x}\right)$$

where σ(*x*) shows output as either 0 or 1 and *e* is the base of the natural log and *x* represents the input. For linearly separable data LR is the best choice. It works well to deal with binary classification problems.

#### 2.2.5. Support Vector Machine

A common supervised learning technique used for classification and regression issues is SVM [39]. The dataset is divided using SVM by creating decision paths known as hyperplanes. SVM can effectively handle both linear and nonlinear data. Because the hyperplane separates the dataset into two groups, linear SVM handles the separable data. Data points above the hyperplane are classified as class 1, while those below the hyperplane are classified as class 2. There are support vectors as well. The points that are near the hyperplane are known as support vectors. SVM separates the data on the one-vs-all concept which stops when the dataset separates into several classes. Nonseprable data is handled by the nonlinear SVM. In non-linear SVM the actual coordinate space is converted to separable coordinate space *x* = ϕ(*x*).

#### 2.2.6. Gradient Boosting Machine

GBM is utilized for both classification and regression issues [40,41]. The main reason for boosting GBM is to enhance the capacity of the model in such a way as to catch the drawbacks of the model and replace them with a strong learner to find the near-to-accurate or perfect solution. This stage is carried out by GBM by gradually, sequentially, and additively training a large number of models. GBM is very sensitive to noisy data. Due to the boosting technique in GBM, it is less susceptible to overfitting problems.

#### 2.2.7. Extra Tree Classifier

ETC is a tree-based learning model that uses the results of multiple correlated DTs for the final prediction [42]. The training samples are used to generate each DT in the forest that will be utilized for further classification. Numerous uncorrelated DTs are constructed using random samples of features. During this process of constructing a tree, the Gini index is used for every feature, and feature selection is performed for data splitting.

#### 2.2.8. Gaussian Naive Bayes

The GNB method is based on the Bayes theorem and assumes that each feature in the model is independent [43,44]. It is used for object classification using uniformly distributed data. It is also known as the GNB classifier because of these features. It can be calculated using the following formula

(7) $$P\left(c|x\right)=\frac{P(c|x)P(c)}{p(x)}$$

(8) $$P\left(c|x\right)=P\left(x_{1}|x\right)*....,P\left(x_{1}|x\right)*P\left(c\right)$$

#### 2.2.9. Stochastic Gradient Decent

SGD integrates many binary classifiers and has undergone extensive testing on a sizable dataset [45,46]. It is easy to develop and comprehend, and its functioning resembles the regression technique quite a bit. SGD hyperparameter settings need to be correct in order to obtain reliable results. The SGD is sensitive to feature scaling.

### 2.3. Convolutional Neural Network for Feature Engineering

In this study, a CNN was used for feature engineering [47,48]. The embedding layer, flatten layer, max-pooling layer, and 1D convolutional layer are the four layers that make up CNN. In this study, an embedding layer with an embedding size of 20,000 was used. This layer utilized the features from the brain tumor dataset. The embedding layer had an output dimension of 300. After this layer 1D convolutional layer was used with a filter size of 5000. ReLU was utilized as an activation function and had a kernel size of 2 × 2. In order to map key features from the output of the 1D convolutional layer, a 2 × 2 max-pooling layer was utilized. The output was flattened at the end, and the ML models were then converted back to 1D arrays. Let ( $fs_{i}$, $tc_{i}$) be a tuple set of brain tumor data set where the target class columns are represented by the tc, the feature set is represented by the fs, and the tuple index is represented by the *I*. An embedding layer was utilized to get the desired output from the training set.

(9) EL=embedding_layer(Vs,Os,I)

(10) EOs=EL(fs)

where $EO_{s}$ denotes the embedding layer outputs and is fed to the convolutional layer as input, the embedding layers are denoted by EL. Three parameters are available in EL: $V_{s}$ as the size of the vocabulary, *I* as the length of the input, and Os as the dimension of the output.

For brain tumor detection, we set the embedding layer at 20,000. This shows that this layer has the ability to take inputs ranging from 0 to 20,000. The length of the input was set at 13 and the output dimension was set at 300. All the input data in the CNN were processed in the embedding layer which created the output for the models for the next processing. The output dimensions of the embedding layers are

(11) 1D−Convs=CNN(F,Ks,AF)←EOs

where the 1D−Convs represents the output of 1D convolutional layers.

For brain tumor detection, we used 500 filters for the CNN i.e., F=500 and the Kernel size is Ks=2×2. The activation function not only changes the negative values but also helps to keep other values unchanged.

(12) f(x)=max(0,E)s

For significant feature mapping the max-pooling layer was utilized in CNN. For brain tumor detection a 2 × 2 pool was used to map the features. Here Fmap represents the features obtained from max-pooling, S-2 shows the stride and Ps=2 is the pooling window size.

(13) $$Cf=Fmap=\lfloor \left(1-P_{s}\right)/S\rfloor +1$$

**Figure 1:**
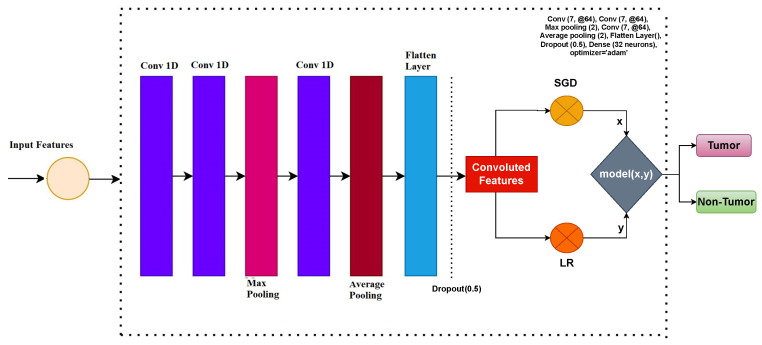
Architecture diagram of the CNN with voting classifier (LR+SGD) model.

A flattened layer was used to transform the 3D data into 1D. The main reason behind this conversion is that the machine learning models work well on the 1D data. For the training of the ML models, the above-mentioned step was implemented and for the training, we obtained the 25,000 features. The architecture of the used CNN along with the predictive model is shown in Figure 1.

### 2.4. Proposed Voting Classifier

For obtaining better results, several studies preferred ensemble machine learning models. When compared with individual models, the performance of ensemble classifiers is better. Therefore, this study used an ensemble model to detect brain tumors.

**Figure 2:**
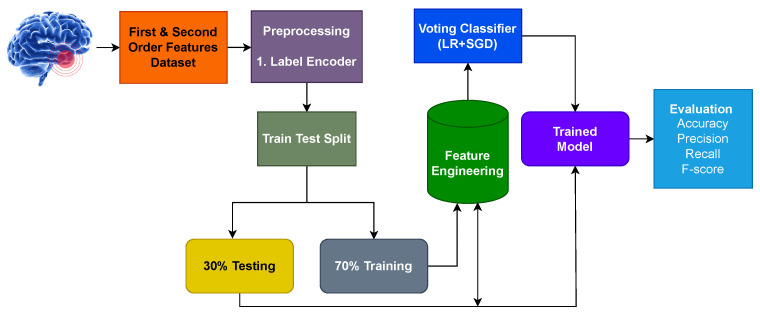
Workflow diagram of the proposed voting classifier (LR+SGD) model.

Figure 2 displays the pipeline flowchart for detecting brain tumors. Two machine learning models, LR and SGD, were combined to create the proposed model. The brain tumor dataset from the Kaggle was used for experiments. The proposed model was used for the brain tumor dataset for two scenarios. Firstly, all 13 features of the brain tumor dataset were used for brain tumor prediction. In the second experiment, the dataset’s characteristics were extracted using CNN, and models were trained on them to distinguish between patient groups with and without tumors. The split of the data is 0.7 to 0.3, with 70% of the data utilized for training and 30% for testing. Accuracy, precision, recall, and F1 score were used to evaluate the model.

**Figure 3:**
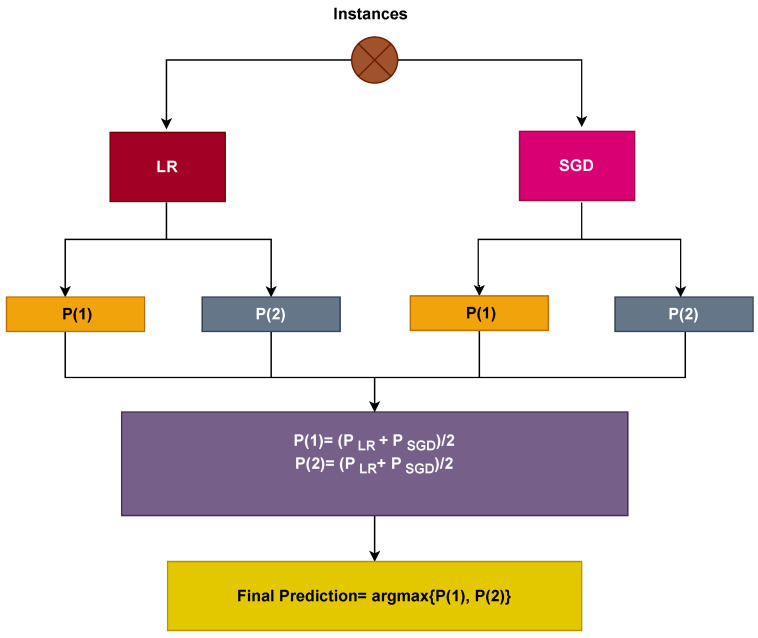
Architecture of the proposed voting classifier (LR+SGD) model.

In this work, LR and SGD are combined with soft voting criteria. The architecture of the voting classifier is given in Figure 3. The outcome with high probability is regarded as the final output in soft voting.

Mathematically, the soft voting criteria can be represented as

(14) $$\hat{p}=argmax\sum_{i}^{n}LR_{i},\sum_{i}^{n}SGD_{i}$$

where the probability values against the test sample are denoted by $\sum_{i}^{n}LR_{i}$ and $\sum_{i}^{n}SGD_{i}$. The probability values for each instance using LR and SGD are then passed through on the basis of soft voting as shown in Figure 3.

Each sample that has passed through the LR and SGD is given a probability score. For example, if the LR model’s probability value is 0.4 and 0.7 for two classes, respectively, and the SGD model’s probability value is 0.5 and 0.4 for two classes, respectively, and P(x) represents the probability value of *x* ranging from 0 to 1, the final probability is determined as

P(1)=(0.4+0.5)/2=0.45

P(2)=(0.7+0.4)/2=0.55

The final output will be 2 because it has the highest probability. By combining the projected probabilities from both classifiers, VC(LR+SGD) selects the final class based on the maximum average probability for each class. The hyperparameter details of all models used in this research work are listed in Table 1.

### 2.5. Evaluation Metrics

Accuracy, precision, recall, and F1 score are the performance metrics utilized in this study to assess the machine learning models’ effectiveness. These measurements are all dependent on the confusion matrix’s values.

(15) $$\begin{array}{c} \mathrm{Accuracy}=\frac{TP+TN}{TP+TN+FP+FN} \end{array}$$

(16) $$\begin{array}{c} \mathrm{Precision}=\frac{TP}{TP+FP} \end{array}$$

(17) $$\begin{array}{c} \mathrm{Recall}=\frac{TP}{TP+FN} \end{array}$$

(18) $$\begin{array}{c} F1\;\mathrm{score}=2\times \frac{\mathrm{Precision}\times \mathrm{Recall}}{\mathrm{Precision}+\mathrm{Recall}} \end{array}$$

## 3. Results and Discussion

### 3.1. Experiment Setup

Several experiments were conducted for the performance analysis, and the performance of the proposed approach was extensively assessed in comparison to the other learning models. All the experiments were performed using a 7th generation Intel Corei7 machine with Windows 10 operating system. Python language was used for the implementation of the proposed approach and the other learning models. Tensor Flow, Sci-kit learn, and Keras libraries were also used. Experiments were carried out in two situations to evaluate the effectiveness of the proposed technique: using original features from the brain tumor dataset and using CNN features.

### 3.2. Performance of Models Using Original Features

The ML models were applied to the actual dataset in the first set of experiments and the results are shown in Table 2. Results show that the SGD and LR achieved the highest accuracy values of 0.881 and 0.869, respectively among all models. RF received a 0.854 accuracy while the LR+SGD ensemble model attained an accuracy score of 0.845. Tree-based model ETC attained an accuracy score of 0.829 while the GNB showed the worst performance with a 0.769 accuracy score. However, the linear models LR, SGD, and their ensemble outperform when using the original feature set.

When compared to other linear models, the performance of the ensemble model was noteworthy. Individually, LR and SGD performed well on the original feature set and their combination further improved the results. Although the proposed voting ensemble model performed well, the obtained accuracy fell short of existing works and lacked the desired accuracy for brain tumor classification. More experiments were conducted for this purpose using CNN as a feature engineering technique and an ensemble learning model.

### 3.3. Results Using CNN Feature Engineering

In the second set of experiments, the performance of the proposed ensemble model and other models was assessed using CNN as a feature engineering technique to extract features from the dataset. Table 3 presents the results of the models when CNN features were used for model training. Expanding the feature set was the main goal of employing CNN model features, which was anticipated to increase the learning models’ accuracy.

The results show that the proposed voting ensemble model LR+SGD leads the performance of all models applied in this study with an accuracy score of 0.995. The proposed ensemble model performs significantly better improving the accuracy by 0.15 over the original feature set. In the same manner, the results of the individual models have also improved using convoluted features. SGD obtained an accuracy score of 0.987 and the regression-based model LR achieves an accuracy score of 0.989. The tree-based models such as ETC and RF obtain accuracy scores of 0.926 and 0.958, respectively. Probability-based model GNB is again the least performer on the CNN features as well and achieved an accuracy score of 0.866. It is noted that GNB also showed some improvement in results as compared to the original features.

### 3.4. Results of K-Fold Cross-Validation

In order to verify the effectiveness of the proposed model this research work makes use of k-fold cross-validation. Table 4 provides the results of the 10-fold cross-validation. Cross-validation results reveal that the proposed ensemble model provides an average accuracy score of 0.996 while the average scores for precision, recall, and F1 are 0.998, 0.998, and 0.997, respectively.

### 3.5. Performance Comparison with State-of-the-Art Approaches

The results of the proposed model are compared with existing state-of-the-art studies to show the performance comparison in Table 5. For this purpose, several recently published works are selected so as to report the most recent results. Ref. [25] uses the NGBoost model for brain tumor detection and obtains 0.985 accuracy. Similarly, the study [26] utilizes a CNN deep learning model for the same task and reports a 0.950 accuracy score with the same dataset used in this study. An EfficientNet-B0 is employed in [27] for brain tumor detection that obtains a 0.988 accuracy score. The current study took the benefit of CNN features to train a voting classifier for brain tumor detection and obtained better results than existing state-of-the-art approaches with a classification accuracy of 0.999.

## 4. Conclusions and Future Work

The goal of this study was to create a framework that can properly distinguish between brain images with and without tumors and minimize the risks associated with this leading cause of mortality. The proposed method focuses on improving accuracy while reducing prediction errors for brain tumor detection. The experimental finding showed that by employing convolutional features, more accurate results were achieved than by using the original features. Furthermore, the ensemble classifier comprising LR and SGD outperformed individual models. Compared with state-of-the-art methods, the proposed method achieved an accuracy score of 0.999, demonstrating its superiority over existing methods and highlighting the effectiveness of the framework. In the future, we intend to employ deep-learning ensemble models to conduct tumor-type classifications with convolutional features. This study used a single dataset obtained from a single source. In the future, we plan to apply the proposed approach to other datasets to demonstrate its generalizability.

## Figures and Tables

**Table 1:** Hyperparameter values of all models used in this research work.

Classifiers	Parameters
RF	number of trees = 200, maximum depth = 30, random state = 52
DT	number of trees = 200, maximum depth = 30, random state = 52
k-NN	algorithm = ‘auto’, leaf size = 30, metric = ‘minkowski’, neighbors = 5, weights = ‘uniform’
LR	penalty = ‘l2’, solver = ‘lbfgs’
SVM	C = 2.0, cache size = 200, gamma = ‘auto’, kernel = ‘linear’, maximum iteration = -1, probability = False, random state = 52, tol = 0.001
GBM	number of trees = 200, maximum depth = 30, random state = 52, learning rate = 0.1
ETC	number of trees = 200, maximum depth = 30, random state = 52
GNB	alpha = 1.0, binarize = 0.0
SGD	penalty = ‘l2’, loss = ‘log’
CNN	Conv (7, @64), Conv (7, @64), Max pooling (2), Conv (7, @64), Average pooling (2), Flatten Layer(), Dropout (0.5), Dense (32 neurons), optimizer = ‘adam’

**Table 2:** Results of machine learning models using the original features.

Model	Accuracy	Class	Precision	Recall	F1 Score
Voting Classifier LR+SGD	0.845	Tumour	0.865	0.899	0.878
		Non-Tumour	0.748	0.799	0.776
		Micro Avg.	0.824	0.858	0.856
		Weighted Avg.	0.807	0.843	0.825
GBM	0.805	Tumour	0.795	0.818	0.807
		Non-Tumour	0.818	0.818	0.818
		Micro Avg.	0.805	0.819	0.827
		Weighted Avg.	0.808	0.814	0.826
GNB	0.769	Tumour	0.777	0.788	0.777
		Non-Tumour	0.744	0.766	0.755
		Micro Avg.	0.766	0.777	0.766
		Weighted Avg.	0.766	0.777	0.766
ETC	0.829	Tumour	0.806	0.806	0.806
		Non-Tumour	0.815	0.815	0.815
		Micro Avg.	0.805	0.805	0.805
		Weighted Avg.	0.809	0.820	0.811
LR	0.869	Tumour	0.866	0.899	0.877
		Non-Tumour	0.888	0.899	0.888
		M Avg.	0.855	0.902	0.883
		W Avg.	0.855	0.884	0.876
SGD	0.881	Tumour	0.903	0.892	0.893
		Non-Tumour	0.923	0.924	0.922
		Micro Avg.	0.922	0.922	0.911
		Weighted Avg.	0.919	0.919	0.919
RF	0.854	Tumour	0.827	0.858	0.834
		Non-Tumour	0.844	0.806	0.828
		Micro Avg.	0.844	0.844	0.833
		Weighted Avg.	0.833	0.833	0.833
DT	0.829	Tumour	0.806	0.822	0.811
		Non-Tumour	0.805	0.833	0.814
		Micro Avg.	0.807	0.809	0.818
		Weighted Avg.	0.818	0.804	0.804
SVM	0.788	Tumour	0.788	0.800	0.799
		Non-Tumour	0.777	0.788	0.788
		Micro Avg.	0.788	0.799	0.800
		Weighted Avg.	0.788	0.799	0.800
k-NN	0.828	Tumour	0.788	0.822	0.800
		Non-Tumour	0.777	0.811	0.800
		Micro Avg.	0.777	0.811	0.800
		Weighted Avg.	0.799	0.824	0.824

**Table 3:** Machine Learning Models Performance Using CNN as feature engineering.

Model	Accuracy	Class	Precision	Recall	F1 Score
Voting Classifier LR+SGD	0.995	Tumour	0.999	0.999	0.999
		Non-Tumour	0.999	0.999	0.999
		Micro Avg.	0.999	0.999	0.999
		Weighted Avg.	0.999	0.999	0.999
GBM	0.905	Tumour	0.928	0.944	0.926
		Non-Tumour	0.915	0.923	0.914
		Micro Avg.	0.927	0.931	0.924
		Weighted Avg.	0.915	0.935	0.918
GNB	0.866	Tumour	0.877	0.888	0.877
		Non-Tumour	0.844	0.866	0.855
		Micro Avg.	0.866	0.877	0.877
		Weighted Avg.	0.855	0.877	0.866
ETC	0.926	Tumour	0.907	0.903	0.905
		Non-Tumour	0.914	0.918	0.914
		Micro Avg.	0.913	0.913	0.913
		Weighted Avg.	0.900	0.900	0.900
LR	0.989	Tumour	0.966	0.999	0.977
		Non-Tumour	0.988	0.999	0.988
		M Avg.	0.977	0.999	0.988
		W Avg.	0.977	0.999	0.988
SGD	0.987	Tumour	0.985	0.997	0.986
		Non-Tumour	0.999	0.986	0.988
		Micro Avg.	0.988	0.988	0.988
		Weighted Avg.	0.988	0.988	0.988
RF	0.958	Tumour	0.927	0.954	0.935
		Non-Tumour	0.944	0.960	0.952
		Micro Avg.	0.944	0.960	0.952
		Weighted Avg.	0.934	0.954	0.944
DT	0.936	Tumour	0.900	0.928	0.914
		Non-Tumour	0.900	0.934	0.912
		Micro Avg.	0.900	0.900	0.915
		Weighted Avg.	0.914	0.900	0.900
SVM	0.978	Tumour	0.974	0.922	0.955
		Non-Tumour	0.977	0.944	0.944
		Micro Avg.	0.977	0.933	0.944
		Weighted Avg.	0.988	0.955	0.966
k-NN	0.982	Tumour	0.988	0.988	0.988
		Non-Tumour	0.977	0.977	0.977
		Micro Avg.	0.966	0.966	0.966
		Weighted Avg.	0.977	0.977	0.977

**Table 4:** Proposed approach k-fold cross-validation result.

Fold Number	Accuracy	Precision	Recall	F-Score
Fold-1	0.992	0.995	0.994	0.995
Fold-2	0.994	0.996	0.995	0.996
Fold-3	0.996	0.997	0.996	0.997
Fold-4	0.998	0.999	1.000	0.998
Fold-5	0.999	0.999	0.998	0.998
Fold-6	1.000	0.999	0.999	0.998
Fold-7	0.995	0.999	0.996	0.997
Fold-8	0.997	0.998	0.997	0.998
Fold-9	0.997	0.997	0.998	0.998
Fold-10	0.999	0.999	0.999	0.999
**Average**	**0.996**	**0.998**	**0.998**	**0.997**

**Table 5:** Performance comparison with state-of-the-art studies.

Reference	Year	Approach	Accuracy
[25]	2020	NGBoost	0.985
[26]	2021	CNN	0.950
[27]	2022	EfficientNet-B0	0.988
Proposed	2022	CNN features and voting Classifier	0.999

## Data Availability

The datasets can be found by the authors at request.
